# Mitochondria-Targeted Antioxidants: A Step towards Disease Treatment

**DOI:** 10.1155/2020/8837893

**Published:** 2020-12-03

**Authors:** Qian Jiang, Jie Yin, Jiashun Chen, Xiaokang Ma, Miaomiao Wu, Gang Liu, Kang Yao, Bie Tan, Yulong Yin

**Affiliations:** ^1^Animal Nutritional Genome and Germplasm Innovation Research Center, College of Animal Science and Technology, Hunan Agricultural University, Changsha, Hunan 410128, China; ^2^Key Laboratory of Feed Biotechnology, The Ministry of Agriculture and Rural Affairs of the People's Republic of China, Beijing 100081, China; ^3^College of Bioscience and Technology, Hunan Agricultural University, Changsha, Hunan 410128, China; ^4^Laboratory of Animal Nutritional Physiology and Metabolic Process, Institute of Subtropical Agriculture, Chinese Academy of Sciences, Changsha, Hunan 410125, China

## Abstract

Mitochondria are the main organelles that produce adenosine 5′-triphosphate (ATP) and reactive oxygen species (ROS) in eukaryotic cells and meanwhile susceptible to oxidative damage. The irreversible oxidative damage in mitochondria has been implicated in various human diseases. Increasing evidence indicates the therapeutic potential of mitochondria-targeted antioxidants (MTAs) for oxidative damage-associated diseases. In this article, we introduce the advantageous properties of MTAs compared with the conventional (nontargeted) ones, review different mitochondria-targeted delivery systems and antioxidants, and summarize their experimental results for various disease treatments in different animal models and clinical trials. The combined evidence demonstrates that mitochondrial redox homeostasis is a potential target for disease treatment. Meanwhile, the limitations and prospects for exploiting MTAs are discussed, which might pave ways for further trial design and drug development.

## 1. Introduction

Mitochondria, subcellular organelles found in most eukaryotic cells, are responsible for numerous metabolic network processes, including the tricarboxylic acid cycle (TCA cycle), glycolysis, oxidative phosphorylation (OXPHOS), amino acid metabolism, and fatty acid oxidation. Among them, the most important physiological function of mitochondria is to generate ATP by oxidizing nutrients. To participate in adenosine 5′-triphosphate (ATP) production, mitochondria use a complex system interacting with these metabolic network processes, during which free radicals are produced. Generally, mitochondrial ROS production mainly occurs at the site of the electron transport chain located on the mitochondrial inner membrane, and the leakage of electrons from complex I and complex III leads to oxygen consumption and superoxide formation [1]. The mitochondrial redox homeostasis refers to an equilibrium between ROS production and scavenging, which is the basis for mitochondrial function and cell fate determination [2].

At present, it is recognized that many pathological changes are associated with impaired mitochondrial function [3], such as increased accumulation of ROS and decreased OXPHOS and ATP production. Although the production of intracellular ROS is itself an inevitable process, cells have an adaptive defense system to scavenge ROS [4]. However, under most oxidative stress conditions, the endogenous antioxidant system in the cells is not enough to scavenge excess ROS. In that case, the accumulation of ROS will cause oxidative damage to intracellular lipids, DNA, and proteins, thereby accelerating the development of related diseases [5]. In the past decade, research has focused on maintaining redox homeostasis and normal function of mitochondria via antioxidants [6]. Current medical projects are aimed at exploiting drugs that restore mitochondrial function and regulate mitochondrial ROS production [7]. To modulate mitochondrial redox homeostasis, the drug should selectively accumulate in the mitochondria and interact with mitochondrial targets, ultimately maintaining normal cellular functions [8]. Although this mitochondrial targeting strategy is attractive, the clinical applications are hampered by some challenges, such as the poor biological availability and the lack of evidence in animal models and clinical research studies [9]. Several drugs have been applied for clinical trials; however, no drug has been approved by the US Food and Drug Administration (FDA) for mitochondria-targeted treatment.

The present review article is aimed at summarizing experimental data on mitochondria-targeted antioxidants (MTAs) for various disease treatments in different models and clinical trials to present the evidence supporting the therapeutic potential of these MTAs. We specifically focused on brain neurological diseases [10, 11], cardiovascular diseases [12–14], and cancer development [15, 16], all of which are closely associated with oxidative damage and signal activation caused by the excess accumulation of ROS in mitochondria. Meanwhile, the potential MTA applications in disease treatment, their limitations, and prospects for exploiting MTAs are discussed.

## 2. Moving Forward from Nontargeted Antioxidants to MTAs

**Figure 1:**
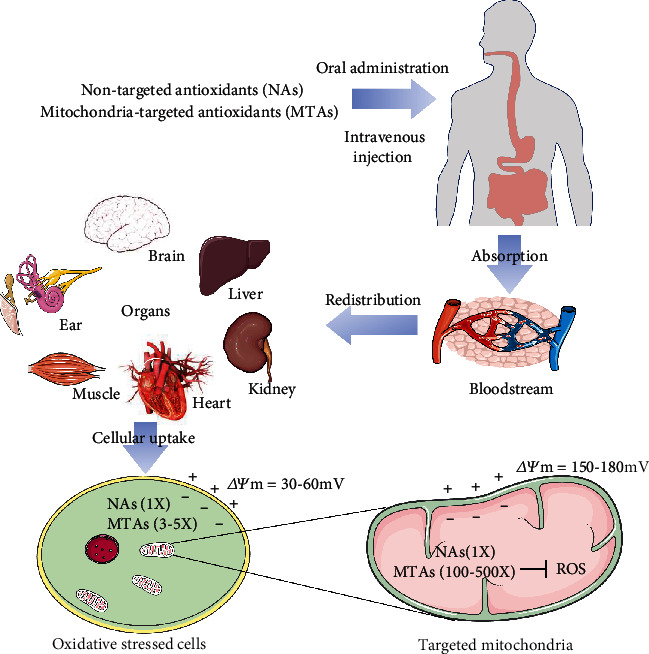
Administration and transport of nontargeted antioxidants (NAs) and mitochondria-targeted antioxidants (MTAs). Ideal antioxidants are bioavailable and can be quickly transported into the blood circulation via intestinal absorption or intravenous injection. The NA can hardly be efficiently delivered to the targeted tissues and mitochondria. The MTAs accumulate 100-500 times in the mitochondria and protect the tissues (brain, liver, kidney, muscle, ear, or heart) from oxidative damage.

An increasing number of studies are aimed at developing conventional (nontargeted) antioxidants for restoring physiology conditions during oxidative stress. Although preliminary studies on many cell or animal models showed promising results, the results from clinical trials were sometimes contradictory. A recent review article [17] has summarized the adverse effects of nontargeted antioxidants (NAs) including vitamin A, vitamin C, vitamin E, and *β*-carotene. These adverse effects of NAs were mainly observed in the treatments of lung cancer and cardiovascular diseases [18]. Redox signaling is an important part of many physiological processes. Excessive or inappropriate use of antioxidants may abolish ROS production and result in compensatory upregulation of mitogen-activated protein kinase (MAPK) pathways [19], which in turn negatively affect the endogenous antioxidant system and normal cell growth [20]. Another concern is whether conventional (nontargeted) antioxidants can be absorbed properly and how they are metabolized in different organs. These uncertainties make it difficult to determine the dose of traditional antioxidants used for disease treatment. The most effective way for an antioxidant stepping forward to disease treatment is to conjugate with a carrier, such as lipophilic cations, liposomes, or peptides, to enable its bioactive ingredient to be targeted for transport into the mitochondria. This targeted delivery enables antioxidants to achieve high concentration accumulation in cells and mitochondria, thereby protecting cells and tissues from oxidative damage through different mechanisms. Ideal antioxidants should be bioavailable and can quickly enter the blood circulation via intestinal absorption or intravenous injection. The MTAs could accumulate in the mitochondria and protect the targeted tissues (brain, liver, kidney, muscle, ear, and heart) from oxidative damage (Figure 1). In the past decade, many studies focusing on the development of mitochondria-targeted antioxidants gave promising results, which we will discuss in detail.

## 3. Lipophilic Cation-Linked MTAs

**Figure 2:**
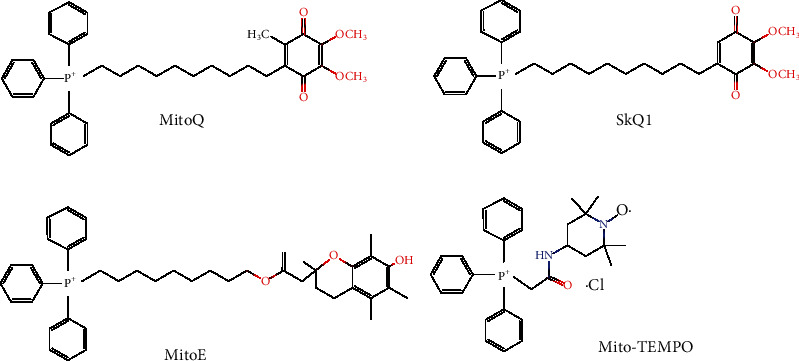
Chemical structures of representative TPP-linked mitochondria-targeted antioxidants (MitoQ, SkQ1, MitoE, and Mito-TEMPO are shown).

The mitochondrial transmembrane potential theory was first proposed by Skulachev et al. in 1969 [21]. The lipophilic cation could easily penetrate cells and mitochondria with the help of *ΔΨ*m, which is positive outside and negative inside. The targeted transport of antioxidants to mitochondria can be achieved by using a lipophilic cation as a transport vehicle. This strategy can be applied for a variety of bioactive substances, especially these hydrophobic ones that are not easily absorbed by cells and mitochondria. In the past decades, triphenylphosphonium (TPP) has been commonly used for the development of MTAs. Presentative studies on the TPP-linked MTAs are summarized in Table 1, and the chemical structures of MitoQ, SkQ1, MitoE, and Mito-TEMPO are shown in Figure 2. Among them, MitoQ and SkQ1 have been extensively studied in various animal models and several human clinical trials [22]. In a clinical trial on twenty healthy older adults (60-79 years) with impaired endothelial function (NCT02597023), oral MitoQ (20 mg/day) supplementation improved brachial artery flow-mediated dilation, decreased aortic stiffness, and lowered the plasma low-density lipoprotein [23]. In a clinical trial on hepatitis C virus- (HCV-) infected patients (NCT00433108), oral MitoQ (40 or 80 mg/day) supplementation decreased serum alanine transaminase (ALT), indicating a decreased necroinflammation in the liver [24]. SkQ1 was documented to relieve the dry eye symptoms in a phase 2 study (NCT02121301) [25]. Meanwhile, a vehicle-controlled study of SkQ1 as a treatment for dry eye syndrome is recruiting (NCT04206020).

Toxicity to mitochondria is a major limiting factor for the application of TPP-linked antioxidants in disease treatment [26]. During the transport of the TPP-linked antioxidants, TPPs increasingly adhere to the surface of the mitochondrial inner membrane. This accumulation of TPPs could destroy the integrity of the mitochondrial membrane and limit aerobic respiration and ATP synthesis [27]. In the toxicity assessment of *in vivo* experiments [28] using a mouse model, the maximum tolerated doses of methyl TPP and MitoE2 are 3.8 and 6.0 mg/(kg∗bodyweight), respectively. Evident toxic effects of TPP and MitoE2 were observed at 6.4 and 10.2 mg/(kg∗bodyweight), respectively. Intravenous injection of MitoQ was not toxic to the mice at 20 mg/(kg∗bodyweight) but significantly toxic at 27.0 mg/(kg∗bodyweight). It is noteworthy that long-term and low-dose MitoQ administration did not exhibit any toxic effect to the mouse models [29], which indicates that the toxic effect is caused by the disruption of normal function of mitochondria in response to a concentrated accumulation of TPP, and an oral administration with low-dose TPP compound is feasible. Therefore, in clinical trials testing TPP-linked antioxidants, it is necessary to strictly control the dosage and to ensure the effective concentration of the MTAs is lower than the threshold that destroys the normal function of mitochondria. Although some of these TPP-linked antioxidants such as MitoQ and SkQ1 have been evaluated in a wide range of clinical trials (NCT03166800, NCT02597023, NCT00329056, NCT03764735, and NCT02121301), more studies are required to assess their optimal dosages for different disease phases, their long-term effects on redox signal activation, and their potential side effects.

## 4. Liposome-Encapsulated Antioxidants

Liposomes are lipid bilayer membrane vesicles first discovered in 1964 and have been commonly used as nanocarriers for pharmaceuticals and bioactive substances [40]. One advantage of the liposomal encapsulation strategy over lipophilic cations is that bioactive molecules can be encapsulated and delivered without altering their molecular structure and bioactivity. The liposome-encapsulated antioxidants are composed of phosphatidylcholine, phosphatidylglycerol, cholesterol, and antioxidant component. The encapsulated antioxidants such as quercetin, N-acetyl-L-cysteine (NAC), and vitamin E exhibited better therapeutic effects on the models of liver injuries [41] and MCF-7 carcinoma cells [42] when compared with those in nonencapsulated form. For example, only liposomal encapsulated NAC can long-lastingly prevent the cytokine-induced neutrophil chemoattractant expression in the lung, thereby protecting the rats against lipopolysaccharide-induced acute respiratory distress syndrome [43]. It has been reviewed that liposomal encapsulated analogs of vitamin E (*α*-tocopheryl succinate and *α*-tocopheryl ether-linked acetic acid) exerted better anticancer effects on various cancer models due to their higher solubility in aqueous solvents [44]. In a clinical study on fatty liver patients, the phospholipid-encapsulated silybin was revealed to protect the liver from oxidative damage via enhancing mitochondrial function and insulin sensitization [45]. Liposome-encapsulated curcumin administration with 100 mg/(kg∗bodyweight) increased the parameters of plasma antioxidant activity in the Sprague-Dawley rat [46]. Likewise, astaxanthin encapsulated within liposomes showed a better bioavailability than the nonencapsulated astaxanthin and ameliorated oxidative parameters in the Sprague-Dawley rat model of lipopolysaccharide- (LPS-) induced acute hepatotoxicity [47].

**Figure 3:**
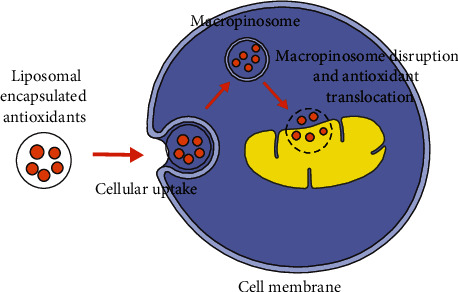
The mitochondrial transport of liposome-encapsulated antioxidants. Liposome-encapsulated antioxidants enter cell membranes via micropinocytosis; after macropinosome disruption, the liposomal components fuse with the mitochondrial membrane, during which the antioxidant components are delivered into the matrix of targeted mitochondria.

Liposome-based delivery systems can carry conventional antioxidants into the mitochondria of the living cells. The transport mechanism of liposome-encapsulated MTAs is shown in Figure 3. Liposome-encapsulated antioxidants enter the cells via micropinocytosis; after macropinosome disruption, the liposomal components fuse with the mitochondrial membrane, during which the antioxidant components are delivered into the matrix of targeted mitochondria. The main disadvantage of the liposome system for MTA delivery is the escape of endosome degradation, which limits the endosomes spontaneously degrading in the cytoplasm and mitochondria. To overcome this limitation, the MITO-Porter that consists of a condensed plasmid DNA and a lipid envelope was developed to deliver bioactive components to mitochondria [48]. The inventors have introduced the characteristics and potential development of MITO-Porter in a specific chapter [49]. Generally, the MITO-Porter-decorated liposomes consist of 1,2-dioleoyl-sn-glycero-3-phosphatidylethanolamine, sphingomyelin, and stearylated octaarginine peptide (R8). During a mitochondria-targeted delivery process, the MITO-Porter-decorated liposomes bind to the mitochondria via electrostatic interactions between R8 and negatively charged mitochondria and then fuse with the mitochondrial membrane. This delivery system can achieve efficient cytoplasmic and mitochondria-targeted delivery, which provides a new way for the treatment of mitochondrial disease. Besides, delivery experiments using fluorescent probes have verified MITO-Porter as an effective tool for macromolecule-targeted delivery [50].

In a mouse model of liver ischemia/reperfusion injury, systemic injection of MITO-Porter-encapsulated CoQ10 (CoQ10-MITO-Porter) decreased serum alanine transaminase (ALT) and prevented kidney injury [51]. Recently, the mitochondrial delivery of methylated *β*-cyclodextrin-threaded polyrotaxanes using a MITO-Porter was revealed to mediate mitochondrial autophagy, which might be useful for mitochondria-associated disease treatment [52]. Moreover, the dual-function MITO-Porter (DF-MITO-Porter) that integrates both R8-modified liposomes and MITO-Porter was developed to effectively deliver exogenous macrobiomolecules into the mitochondria, providing an excellent delivery system for mitochondrial disease treatment [53]. More research studies on the MITO-Porter delivery system are expected to be conducted to shed more light on the mitochondrial therapeutic strategy and targeted antioxidant development.

## 5. Peptide-Based Mitochondrial Antioxidants

The Szeto-Schiller peptide (SS-peptide) and the mitochondria-penetrating peptide (MPP) are peptide chain-based antioxidant delivery systems. SS-peptides contain different small-molecule lipophilic antioxidant compounds and three positive charges and can be targeted-delivered to the mitochondria with the help of *ΔΨ*m of the cellular membrane and mitochondrial membrane [54]. The advantageous properties of SS-peptides include the following: (1) alternating the MPP sequence between the basic and aromatic residues which favor their efficient absorption by cells; (2) unsaturated transport independently from the energy state or a dedicated peptide transporter [55]; (3) small and easily soluble in water, easy to synthesize, and the presence of D-amino acids at specific positions which prevents them from being degraded by aminopeptidases and allows them to be effectively transported into the mitochondria [56]; and (4) 1000-5000 times accumulation in the mitochondria.

Various experiments have confirmed that SS-peptides can be rapidly absorbed by different cell types, such as neurons [57], kidneys [58], epithelial cells, and endothelial cells [59]. It is noteworthy that the mitochondrial uptake speed of SS-peptides is *ΔΨ*m-independent. The absorption of the SS-peptides does not affect the polarization of the mitochondrial membrane, which makes them ideal antioxidants for disease treatment [60]. For example, SS-02 was revealed to easily penetrate a single layer of intestinal epithelial cells from the basal and apical direction [61]. SS-02 has also been reported to penetrate the blood-brain barrier and thus serve as a neuroprotective agent [62]. The SS-peptides are effective in alleviating oxidative stress both in cell models and isolated mitochondria [63], among them SS-31 was widely validated to be effective. The therapeutic potential of SS-31 has been documented for many conditions including brain microvascular endothelial cell damage [64], lateral line hair cell damage [65], mitochondrial morphogenesis [66], atherosclerosis [67], Friedreich ataxia [68], renal fibrosis [69], limb ischemia-reperfusion injury [70], exercise tolerance [71], type 2 diabetes [72], hearing loss [73], neurovascular coupling responses [74], cardiac arrest [75], traumatic brain injury [76, 77], heart failure [78–81], and acute kidney injury [82]. Of importance, the phase 2a clinical trial of SS-31 (unique identifier: NCT01755858) on the atherosclerotic renal artery stenosis patients (ARASP) showed that supplementing with SS-31 during percutaneous transluminal renal angioplasty alleviated the pathological symptoms and improved kidney function, indicating a positive prospect of SS-31 in clinical application for ARASP [83].

A recent study on aged mice revealed that the disruption of mitochondrial redox homeostasis in muscle resulted in energy defect and exercise intolerance, and SS-31 administration restored redox homeostasis of the aged muscle, thereby increasing the exercise tolerance [71]. Five hours of SS-31 treatment significantly decreased mortality of cardiac arrest rats, during which the blood lactate level in the SS-31-treated rats was significantly decreased, suggesting improved mitochondrial aerobic respiration by SS-31 treatment [75]. The antioxidative roles of SS-31 have been also documented in kidney glomerular mitochondria [84]. SS-31 administration was revealed to prevent negative changes in pathological parameters in chronic kidney disease models [69]. More recently, an acute kidney injury- (AKI-) targeted nanopolyplex was designed for SS-31 delivery, which demonstrates a positive effect of combining the use of nanopolyplexes and SS-31 in the oxidative stressed and inflamed kidney [82]. Similarly, treatment with SS-31 was found to decrease cytoplasmic and mitochondrial O^2-^ production by regulating the expression of NADPH oxidase subunit NOX4 in a model of traumatic brain injury [76].

**Figure 4:**
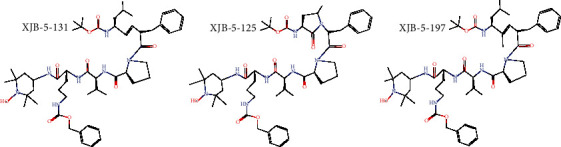
Chemical structures of XJB peptide-based mitochondria-targeted antioxidants (XJB-5-131, XJB-5-125, and XJB-5-197 are shown).

Mitochondria-penetrating peptides (MPPs) consist of 4 to 8 alternating positively charged hydrophobically modified amino acids. They have been widely used for the targeted delivery of mitochondrial small molecules with the help of *ΔΨ*m [85]. A series of XJB peptide-based antioxidants (XJB-5-131, XJB-5-125, and XJB-5-197) have been developed (Figure 4). XJB-5-131, a scavenger for mitochondrial ROS, is the most studied among all the XJB peptide-based antioxidants and has been reported to promote weight gain, prevent neuronal death, and reduce oxidative damage in a mouse model of neurodegeneration [86]. Besides, XJB-5-131 was demonstrated to alleviate oxidative damage of DNA and improve physiology behavior in a Huntington's disease model [87, 88]. Likewise, the compounds of XJB-5-131 and JP4-039 were reported to inhibit ferroptosis via scavenging ROS and altering the subcellular localization of the ferroptosis suppressors [89]. These findings encourage more therapeutic evaluation of XJB peptide-based antioxidants in clinical trials.

## 6. Potential Applications of MTAs in Disease Treatment

It is noteworthy that MTAs may exert multiple effects, such as alterations in redox status, ETC activity, and ATP synthesis during disease treatments, which could be affected by variations of disease types and phases. Proper dosage of the MTAs used in the trials lowers the ROS production in mitochondria and benefits the disease treatments; however, in some cases, a high dosage of MTAs may inhibit ETC activity and promote oxidative damage. Below, we reviewed the experimental and clinical results in Parkinson's disease (PD), traumatic brain injury (TBI), cardiovascular disorders/cardiovascular diseases (CVDs), or cancers, emphasizing the MTA dosage and potential target mechanisms (Tables 2–5).

### 6.1. Parkinson's Disease

Parkinson's disease (PD) is a progressive neurodegenerative disease that mainly occurs in the elderly, without any acknowledged therapies. Evidence from *in vitro* cell models, animal models of PD, and genetic analysis has indicated the involvement of oxidative stress and mitochondrial dysfunction during PD development [90]. Thus, the antioxidative strategy shows great potential for PD therapy. In the past two decades, amounts of studies have been conducted and revealed the beneficial roles of antioxidants in the different cellular and animal models; however, the clinical trials using antioxidants (e.g., NAC (oral 1800, 3600 mg daily; 900 mg effervescent tablet daily), glutathione (100-200 mg daily), and vitamin E (1200 IU/day)+coenzyme Q10 (1200, 2400 mg daily)) to treat PD are mostly disappointing. The experimental factors including inefficient oral administration (NCT01470027, direct oral without any coating or carrier), inadequate patients' replicates (NCT01427517, totally 9 participants were involved; NCT02212678, 8 participants were enrolled), and inappropriate outcome measures (NCT00329056, only UPDRS results were provided; NCT02212678, only GSH levels were provided) may explain these frustrating outcomes from clinical studies. Another possible explanation for this ineffectiveness is that the phase for the antioxidant's treatment is too late for the neurons' rescue. Clinical trial showing high doses of CoQ10 administration benefits the PD patients (NCT01892176), which implies that the traditional antioxidants lack bioavailability, with small scales that can be absorbed into the mitochondria. A systematic review and meta-analysis concluded that CoQ10 cannot provide any symptomatic benefit for PD patients [91]. Consequently, approaches delivering antioxidants to mitochondria for PD treatment have been explored. MitoQ was firstly approved for the clinical trials of PD (NCT00329056) in 2006. A study showed MitoQ (40, 80 mg daily) could slow the progression of PD as measured by the Unified Parkinson's Disease Rating Scale (UPDRS); however, no significant difference between MitoQ and placebo on any measure of PD progression was observed [92]. In regard to dosage, although the experiments *in vitro* (50 nM, 10 *μ*M) and on mouse models (daily 4 mg/kg∗bodyweight) have shown the beneficial effects of MitoQ against mitochondrial dysfunction via preserving striatal dopamine and improving motor functions [93], more study might be conducted to optimize the oral or injection dosage and for the preclinical trials. Besides, the peptide-based mitochondrial antioxidants, such as SS-31 and SS-20 (0.5-5.0 mg/kg∗bodyweight), have shown similar neuroprotective effects on cellular and mouse PD models induced by MPTP [94]; however, the clinical trials using peptide-based mitochondrial antioxidants to treat PD have not been approved until now, which might be hampered by the undesirable results of MitoQ in the clinical trials.

The nanocarrier delivery system that consisted of FDA-approved Pluronic F68 and dequalinium has been revealed to enhance the bioavailability of NAC protecting against the reduced cell viability and oxidative stress in the cellular model of PD, which raises a significant prospect of nanocarrier-based NAC to be transitioned for clinical trials [95]. As indicated by the results from previous clinical trials, the available clinical therapies using antioxidants for PD can only alleviate the symptoms; but none can prevent neuronal degeneration via regulating the dopaminergic system; thus, the combination of MTAs with the traditional drugs (dopamine receptor activator, such as pramipexole) could be a considerable strategy for the further experiments and clinical trials.

### 6.2. Traumatic Brain Injury (TBI)

TBI is a significant cause of death and disability, with an estimated 60-80 million cases per year worldwide, and has been considered an important medical and social problem. TBI can easily damage the cerebral circulatory, which in turn leads to cerebral artery contraction, glutamate poisoning, mitochondrial dysfunction, inflammatory response, and cell death, thereby increasing the severity of the primary damage and causing secondary brain damage [98]. Mechanistically, selective peroxidation of cardiolipin, impaired electron transport, decreased ATP production, and increased formation of ROS during TBI development lead to the final neurodegeneration and brain atrophy [99, 100]. In the past two decades, the beneficial effect of vitamins C and E, progesterone, and NAC to be used for adjuvant therapy in TBI has been evaluated [101]. Nontargeted antioxidants, commonly in very high concentrations, are used to achieve therapeutic effects. It should be noted that mitochondria are the main source of ROS and determine cell fate [102]; antioxidant delivery to mitochondria is an important target for TBI intervention therapy.

N-Acetylcysteine (NCT00822263), docosahexaenoic acid (NCT01903525), and melatonin (NCT04034771) had been approved for the clinical TBI trials. These clinical results indicated that the NAC (4 grams daily) administration could reduce the sequela of mild TBI [103]. Although the measurements of temperature, mean arterial pressure, intracranial pressure (ICP), use of ICP-directed therapies, surveillance serum brain injury biomarkers, and Glasgow Outcome Scale (GOS) at 3 months were not different between the NAC group and the placebo group [104], the metabolomic results support the antioxidative therapeutic target by the probenecid and N-acetylcysteine treatment [105]. Compared with nontargeted antioxidants, relatively low concentrations of MTAs show higher antioxidant activity. The antioxidant activity of SkQR1, XJB-5-131, Mito-TEMPO, SS-31, and MitoQ was revealed in TBI models (Table 3). Although the MTA dosages and mechanisms involved in the therapeutic efficiency are different, data from these different experimental models suggest that MTAs may be a more effective means for mitigating the negative effects of TBI. In terms of mechanism, in addition to antioxidant efficiency, the activation of anti-inflammatory and Nrf2-ARE signaling may also be key indicators for the effectiveness evaluation of MTAs. Until now, the clinical trials using MTAs to treat TBI have not been approved. In future studies, while focusing on the action mechanism and effective dose of various MTAs, the possible toxicological properties of these MTAs also need to be clarified.

### 6.3. Cardiovascular Diseases (CVDs)

Cell redox homeostasis maintains a healthy physiological state of cardiomyocytes and vascular endothelial cells. The content of superoxide anions in the failing human myocardium was found to be twice more than that in the healthy myocardium [111]. There are also similar observations in diabetes [112] and hypertensive cardiomyopathy [113]. Besides, the damage parameters of lipid oxidative, nucleic acid, and protein have been observed in the circulation or myocardial tissue of patients with myocardial infarction or heart failure in the animal models of these conditions [113–116]. Moreover, the oxidative damage of the mitochondria and ROS production by endothelial cells were significantly higher compared with the myocardium of young mice, indicating oxidative stress is also involved in age-related CVDs. The depletion of Sod2 (encoding mitochondrial superoxide dismutase) can aggravate the atherosclerotic process in mice, which confirms the detrimental role of ROS overproduction [117]. Interestingly, the absence of a specific cardiomyocyte Txnrd2 (encoding thioredoxin reductase 2) results in fatal dilated cardiomyopathy in the mouse embryo [118]. These observations indicate that overloaded oxidative stress is associated with a variety of cardiovascular diseases, such as atherosclerosis, cardiac hypertrophy, cardiomyopathy, and heart failure [119].

In the cases of overloaded oxidative stress, a timely supplement of antioxidants to maintain the normal function of the cardiovascular system is crucial for the prevention and treatment of cardiovascular diseases. Some conventional antioxidants such as CoQ10 [120], polyphenols [121], vitamin C [122], and vitamin E [123] have been shown to prevent and treat cardiovascular disease in models, with expanded ongoing clinical trials (NCT03133793, NCT01925937, NCT02779634, NCT02847585, NCT02934555, and NCT02218476). However, the majority of these clinical studies are in unknown status which might be attributed to the poor biopharmaceutical properties and the pharmacokinetics of the nontargeted antioxidants. Mitochondria are the main sites for the ROS production and oxidative energy metabolism, implying the MTAs might show better efficacy for the treatment of cardiovascular disease. In recent studies, promising results have been obtained with the MTAs, including Mito-TEMPO, MitoSNO, SkQ1, MitoQ, MitoE, SS-20, and SS-31, in the cellular and animal models (Table 4), fostering the initiation of clinical trials for CVD treatment. These promising results from preclinical experiments of MitoQ have fostered ongoing clinical trials for diastolic dysfunction (NCT03586414, suspended due to COVID-19 outbreak) and peripheral artery disease (NCT03506633, under recruiting). Clinical trials of SS-31 (formerly named as Bendavia or MTP-131) on the treatment of reperfusion injury (NCT01572909) and heart failure (NCT02388464, NCT02788747, NCT02245620, and NCT02814097) have been completed. The clinical results on reperfusion injury patients indicated that SS-31 combination therapy is superior to placebo [124]. These clinical trials on heart failure patients revealed that SS-31 brings favorable changes in left ventricular volumes in a dose-dependent manner. Recently, one study using an explanted human heart tissue model extended these clinical trials and revealed the beneficial effects of SS-31 on mitochondrial function in heart failure [78]. For further application, the clinical dosage and therapeutic effects of MTAs on the treatment of CVDs need to be further explored.

### 6.4. Cancer

The mitochondria-derived ROS are crucial in cancer development, which makes mitochondria-targeted antioxidants promising anticancer agents. The relationship between mitochondrial ROS and cancer development has been reviewed in recent publications [134, 135]. Generally, changes in the mitochondrial function of tumor cells (“aerobic glycolysis,” also known as “Warburg effect”) lead to further mitochondrial ROS production and nuclear DNA mutations that impair OXPHOS. As a consequence, oncogenic ROS promote the occurrence and development of tumors via inducing DNA damage and regulating various signaling pathways. For example, the production of H_2_O_2_ mediated by endogenous oncogenes can improve the proliferation rate of tumor cells via regulating MAPK signals and stimulating extracellular ERK pathway kinase. Mitochondrial production of O^2-^ stimulates the growth of KRAS lung cancer cells through MAPK/ERK signaling [136]. The upregulation of ROS also activates transcription factors such as nuclear factor-*κ*B (NF-*κ*B), which increases the proliferation of cancer cells [137].

**Figure 5:**
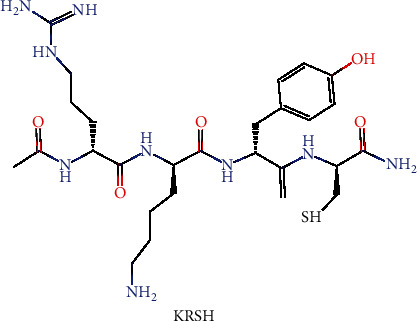
Chemical structure of KRSH.

In the past two decades, several studies focused on targeting mitochondria for anticancer therapy. The anticancer mechanism of these agents includes the following: (1) increasing the conductivity of mitochondrial transition pore complex (PTPC), thereby promoting the rupture of mitochondrial membrane and the release of mitochondrial apoptotic factors [138]; (2) targeting proapoptotic Bcl-2 homology domain 3 (BH3) protein mimetics, during which the apoptotic factors are released [139]; (3) sensitizing the cancer cells to conventional treatments via inhibiting glycolysis of cancer cells [140]; and (4) interrupting glutamine catabolism, pyruvate dehydrogenase, and lactate dehydrogenase [138]. Comparatively, the MTAs with the properties of scavenging the mitochondrial ROS or inhibiting the ROS production could only inhibit the proliferation of cancer cells instead of triggering apoptosis. Several preclinical studies have shown the antigrowth effects of MTAs on cancer models (Table 5). Among the MTAs used for the preclinical experiments, the SkQ1 was the most promising and extensively studied one showing anticancer effects at a nanomole dosage in various models. For example, 40 nmol/L SkQ1 treatment suppressed the proliferation of HT1080 and RD tumor cells in culture via inhibiting mitosis [141]. Daily 5, 30, or 50 nmol/kg∗bodyweight SkQ1-supplemented diet decreased the incidence of spontaneous cancers in p53 knockout mice, BALB/c mice, and benzopyrene-induced mice, respectively [142–144]. Recently, a mitochondria-targeted peptide KRSH that consisted of lysine, arginine, tyrosine, and cysteine (Figure 5) was revealed to inhibit cell proliferation and increase apoptosis of HeLa and MCF-7 cell lines [145]. Mechanically, the positively charged lysine and arginine help the KRSH mitochondria-targeted delivery, meanwhile, the tyrosine and cysteine play antioxidative roles for scavenging mitochondrial ROS. The possible proapoptotic effects of KRSH on HeLa and MCF-7 cells might be attributed to the increased mitochondrial depolarization, but not the antioxidative effects, although the definite mechanism is unknown. The Mito-TEMPO, a TPP-linked MTA whose chemical structure is shown in Figure 2, was revealed to increase the survival ratio and decrease the tumor incidence and tumor multiplicity in the N-nitrosodiethylamine-induced hepatocarcinogenesis mice [146]; however, no *in vitro* evidence attributes its anticancer effect to the ROS scavenging.

MTAs have yielded promising results in several *in vitro* and animal models for cancer studies; however, it is noteworthy that some subsets of cancer cells, such as melanoma tumor cells, exhibit metabolic reprogramming heterogeneity, showing different bioenergy and ROS detoxification capabilities [147]. Besides, a study comparing the effects of MTAs and NAs on the hepatocarcinogenesis indicates contradictory results; that is, nontargeted antioxidants (NAC and vitamin E analog Trolox) prevented tumorigenesis, whereas MTAs (SS-31 and Mito-Q) aggravated tumorigenesis [148]. Therefore, it is critical to clarify the metabolic patterns of different cancer cells in a specific stage and to carefully adopt appropriate therapeutic strategies before clinical intervention with MTAs.

## 7. Conclusion

Mitochondria produce most of the energy and ROS in cells. Mitochondrial ROS are important signaling molecules involved in many cellular adaptative oxidative defense systems. However, excessive ROS accumulation or insufficient clearance results in damaged mitochondrial DNA and protein, both of which are pathophysiological features of a variety of diseases. In the past decades, many studies focused on developing NAs to restore the normal physiological function of oxidative stressed mitochondria. Research studies on various models were promising, but clinical trials sometimes showed contradictory results. Redox signaling is an important part of many physiological processes. Excessive or inappropriate use of antioxidants may abolish ROS production and result in compensatory upregulation of MAPK pathways, which in turn break down the endogenous antioxidant system. Thus, applying the appropriate dosage and delivery method of these antioxidants to balance ROS production and antioxidation is crucial for the clinical trials. Recently, a variety of mitochondria-targeted delivery systems and antioxidants have been exploited to recover mitochondrial function from the pathological conditions in different mechanisms. The outstanding advantages of MTAs over the nontargeted ones include (1) efficient pharmacokinetics and absorption and (2) specific accumulation at cells and mitochondria, avoiding nonspecific high concentration-induced side effects.

This article reviews the characteristics and applications of different mitochondria-targeting tools, including lipophilic cations, liposome vectors, peptide-based targeting, and their recent research reports. Overall, most of these tools have shown beneficial roles for mitochondria-targeting delivery. Based on these delivery tools, an increasing number of MTAs are currently being evaluated, some of which have been validated as effective agents in stage 2 clinical trials, providing unlimited possibilities for mitochondria-targeted therapies. Although the results from current studies are very promising, the human clinical trials on different disease stages should be firstly standardized to effectively translate these research results into usable medicines. Besides, these noteworthy questions should be preferentially considered to exploit more MTAs for disease treatment in the future (refer to noteworthy questions).

## Figures and Tables

**Table 1:** TPP-linked MTAs.

Mitochondria-targeted antioxidants	Bioactive component	Linker	Effects	Reference
MitoE	Vitamin E	2-Carbon aliphatic linker	(1) Minimized lipid peroxidation and protected cells from oxidative damage (2) Eliminated H_2_O_2_-induced oxidative stress and caspase activation in cells (3) Accumulated in tissues (heart, brain, muscle, liver, and kidney) and protected tissues from oxidative damage	[28, 30]
Mito-vitamin E derivation	Vitamin E	11-Alkyl linker	(1) Inhibited energy metabolism and promote cell death (2) Antitumor properties	[31, 32]
SkQ1 SkQR1	Plastoquinone	10-Alkyl linker	(1) Minimized lipid peroxidation and ROS-induced apoptosis (2) Beneficial roles in many diseases including aging, stroke, myocardial infarction, sarcopenia, dry eye syndrome, vascular inflammation	[33, 34]
MitoQ	Coenzyme Q	10-Alkyl linker	(1) Penetrated the mitochondrial membrane and inhibited lipid peroxidation (2) Beneficial roles in animal models of alcoholic fatty liver, neurodegenerative diseases, ischemia-reperfusion, hypertension, sepsis, and kidney damage in type I diabetes	[35, 36]
MitoC MitoVitC_11_	Vitamin C	Thioalkyl linker	(1) Prevented mitochondrial lipid peroxidation and protected mitochondrial aconitase (2) Scavenged O^2–^, peroxyl radicals, and Fe^3+^ and could be rapidly recycled to the active ascorbate moiety	[37]
MitoSOD	M40403	Thioalkyl linker	(1) Regulated the mitochondrial redox system to convert ROS (2) Reversed the rapid and progressive inhibition of aconitase through redox cycling (3) Retained Mn^2+^ under nonacidic conditions	[38, 39]

Notes: *ΔΨ*m: mitochondrial membrane potential; M40403: a macrocyclic Mn SOD mimetic system; ROS: reactive oxygen species; TPP: triphenylphosphonium.

**Table 2:** MTAs in PD models and clinical trials.

Mitochondria-targeted antioxidants	Models/clinical trials	Dosage	Effects/mechanism	Reference
MitoQ	Cellular MPP^+^ model	50 nmol/L in culture medium	(1) Inhibited MPP^+^-induced decrease in dopamine levels	[93]
	Mouse MPTP model	4 mg/kg∗bodyweight; oral gavage	(1) Protected the nigrostriatal axis against MPTP toxicity (2) Improved locomotor activities in MPTP-treated mice (3) Inhibited mitochondrial aconitase inactivation	[93, 96]
	Cellular 6-OHDA model	10-200 nmol/L in culture medium	(1) Blocked 6-OHDA-induced mitochondrial fragmentation	[97]
	Mouse 6-OHDA model	5 mg/kg∗bodyweight; intragastric administration	(1) Rescued dopamine neurons loss in SNc (2) Protected dopamine neurons via activating PGC-1*α* and enhance Mfn2-dependent mitochondrial fusion	[97]
	Clinical trial	Daily 40/80 mg; oral administration	(1) Slowed the progression of Parkinson's disease as measured by the UPDRS (2) No difference in the measured parameters between the treatment and the placebo	NCT00329056

SS-20/Phe-D-Arg-Phe-Lys-NH_2_	Cellular MPP^+^ model	1-10 nmol/L in culture medium	(1) Rescued mitochondrial oxygen consumption and ATP production damaged by MPP^+^ (2) SS-20 (4 mg/kg∗bodyweight) protected against the loss of dopaminergic neurons in the substantia nigra pars compacta	[94]
	Mouse MPTP model	0.5-5 mg/kg∗bodyweight; intraperitoneal injection		

SS-31/D-Arg-(2′6′-dimethyltyrosine)-Lys-Phe-NH_2_	Cellular MPP^+^ model	1-10 nmol/L in culture medium	(1) Improved cell survival and motor performance (2) Decreased cell loss and oxidative stress in the lumbar spinal cord (3) SS-31 (10 mg/kg∗bodyweight) protected against the loss of dopamine and its metabolites	[94]
	Mouse MPTP model	0.5-10 mg/kg∗bodyweight; intraperitoneal injection		

P68+DQA nanocarriers NAC	Cellular rotenone PD model	1000 *μ*mol/L in culture medium	(1) P68+DQA nanocarrier delivery system enhanced the stability, bioavailability, and brain penetrance of NAC (2) Formulation of NAC into P68+DQA nanocarriers rescued cell viability and alleviated oxidative stress	[95]

Notes: 6-OHDA: 6-hydroxydopamine; DQA: dequalinium; Mfn2: mitochondrial GTPase mitofusin-2; MPP^+^: 1-methyl-4-phenylpyridinium; MPTP: 1-methyl-4-phenyl-1,2,3,6-tetrahydropyridine; NAC: N-acetylcysteine; P68: Pluronic F68; PD: Parkinson's disease; PGC-1*α*: peroxisome proliferator-activated receptor gamma coactivator 1 alpha; SNc: substantia nigra pars compacta; UPDRS: Unified Parkinson's Disease Rating Scale.

**Table 3:** MTAs in TBI models.

Mitochondria-targeted antioxidants/bioactive component	Models/clinical trials	Dosage	Effects/mechanism	Reference
SkQR1	Rat model by brain surgery	100 nmol/kg; intraperitoneal injection	(1) Decreased the neurological deficit (2) Lowered the volume of the lesion in the brain cortex (3) Decreased mitochondrial ROS and GSK-3*β* activity	[106]
	Rat model of focal one-sided TBI	250 nmol/kg; intraperitoneal injection	(1) Rescued the disruptions of limb functions (2) Increased survivability of neurons (3) Decreased astroglial expression and infiltration with segmented neutrophils (4) Beneficial effects are dependent on the reduction of mitochondrial reactive oxygen species	[107]

XJB-5-131	Rat CCI model after TBI	10 mg/kg bodyweight; intravenous injection	(1) Protected brain thiols, GSH and PSH, oxidized by TBI (2) Decreased caspase 3/7 activity and attenuated apoptotic neuronal death (3) Scavenged the electrons leaking from electron carriers	[108]

Mito-TEMPO	Isolated MCAs from rats with traumatic injury	30 nmol in the vessel chamber	(1) Alleviated myogenic constriction (2) Scavenged H_2_O_2_ (PEG-catalase) by blocking both BKCa channels and TRPV4 channels	[109]

SS-31	Marmarou's weight drop model of TBI	5 mg/kg; intraperitoneal administration	(1) Rescued mitochondrial dysfunction, and alleviated secondary brain injury (2) Decreased ROS, malondialdehyde, and cytochrome c release and prevented the decline of SOD activity (3) Attenuated neurological deficits, brain water content, DNA damage, and neural apoptosis	[77]

MitoQ	Marmarou's weight drop model	4 mg/kg; intraperitoneal administration	(1) Alleviated neurological deficits and brain edema and inhibited cortical neuronal apoptosis (2) Increased the activity of SOD and GPx and decreased MDA level (3) Reduced Bax translocation to mitochondria and cytochrome c release into the cytosol (4) Accelerated the Nrf2 nuclear translocation and upregulated the Nrf2 downstream proteins, including HO-1 and Nqo1	[110]

Notes: Bax: (Bcl-2)-associated X; BKCa: big conductance Ca^2+^-activated K^+^; CCI: chronic constriction injury; GPx: glutathione peroxidase; GSH: glutathione; GSK-3*β*: glycogen synthase kinase-3*β*; HO-1: heme oxygenase-1; MCAs: middle cerebral arteries; MDA: malondialdehyde; Nqo1: quinone oxidoreductase 1; Nrf2: nuclear factor erythroid 2; PEG-catalase: polyethylene glycol; PSH: protein thiols; SOD: superoxide dismutase; TBI: traumatic brain injury.

**Table 4:** MTAs in cardiovascular disease (CVD) models.

Mitochondria-targeted antioxidants/bioactive component	Models/clinical trials	Dosage	Effects/mechanism	Reference
Mito-TEMPO	THP-1 cell model induced by ox-LDL; high-fat dietary-fed rats	20 *μ*mol/L; 0.7 mg/kg∗bodyweight; intraperitoneal administration	(1) Attenuated foam cell formation via promoting autophagic flux (2) Increased cholesterol efflux via autophagy-dependent ABCA1 and ABCG1 upregulation (3) Reversed the accumulation of TC and LDL-c	[125]

MitoSNO	Open chest mouse model	100 ng/kg∗bodyweight; intravenous injection	(1) Reduced infarct size and troponin release (2) Ineffectiveness on hemodynamics in the heart, *dP*/*dt*_max_ or heart rate (3) Alleviated infarction and myocardial fibrosis	[126]

SkQ1	Lifelong treatment of mice	1 or 30 nmol/kg∗bodyweight	(1) Prevented spontaneous cardiomyopathy (2) Decreased age-related heart hypertrophy and diffuse fibrosis (3) Affected cell adhesion-related gene expressions, one of which had mitochondrial localization	[127]

MitoQ	Pressure overload-induced heart failure in rats	100 *μ*mol/L in drinking water	(1) Reduced ventricular hypertrophy and lung congestion (2) Restored membrane potential in IFM (3) Improved retention capacity of mitochondrial calcium in the SSM and IFM	[128]
	Pressure overload-induced cardiac fibrosis in rats	2 *μ*mol; oral gavage	(1) Attenuated apoptosis, hypertrophic remodeling, fibrosis, and left ventricular dysfunction (2) Blunted TGF-*β*1 and NOX4 upregulation (3) Prevented Nrf2 downregulation and rescued TGF-*β*1 activation (4) Ameliorated the cardiac remodeling dysregulation in phenylephrine and TGF-*β*1-induced models	[129]
	Rat model of prenatal hypoxia	125 *μ*mol; intravenous injection	(1) Improved vasorelaxation (2) Alleviated oxidative stress in placental cells (3) Prevented the decrease in vascular sensitivity to phenylephrine of their offspring	[130]
	Mouse model of aortic stiffening	250 *μ*mol/L in drinking water	(1) Decreased pulse wave velocity in old mice (2) Rescued the decrease of elastin region elastic modulus and elastin expression (3) Reversed *in vivo* aortic stiffness	[131]

MitoE	Bovine aortic endothelial cells induced by hydrogen peroxide and glucose oxidase	1 *μ*mol/L in culture medium	(1) Abrogated H_2_O_2_- and lipid peroxide-induced oxidative protein (2) Inhibited cytochrome c release, caspase 3 activation, and DNA fragmentation (3) Inhibited transferrin receptor-dependent iron uptake and apoptosis	[132]

SS-20, SS-31	Rat model of myocardial infarction	3 mg/kg∗bodyweight; intraperitoneal injection	(1) Reduced lipid peroxidation (2) Decreased the occurrence frequency and severity of arrhythmia	[133]

SS-31/elamipretide/MTP-131	Clinical trials on heart failure patients	20 mg subcutaneous injection 4 and 40 mg intravenous injection	(1) High-dose SS-31 improved left ventricular volumes (2) Improved super complex-associated oxygen flux, complex (C) I activity	NCT02388464 [78]
	Clinical trials on reperfusion injury patients	Intravenous at 0.05 mg/kg/h	(1) Conjunction SS-31 with standard therapy is superior to placebo for reducing myocardial infarction	NCT01572909 [124]

Notes: CF: cardiac fibroblasts; IFM: interfibrillar mitochondria; LDL-c: high-density lipoprotein cholesterol; lncRNAs: long noncoding RNAs; NOX4: NADPH oxidase subunit 4; Nrf2: nuclear factor erythroid 2; ox-LDL: oxidized high-density lipoprotein; SSM: subsarcolemmal mitochondria; TC: total cholesterol; TEMPO: 4-hydroxy-2,2,6,6-tetramethylpiperidin-N-oxide; TGF-*β*1: transforming growth factor *β* 1.

**Table 5:** MTAs in cancer models.

Mitochondria-targeted antioxidants/bioactive component	Models/clinical trials	Dosage	Effects/mechanism	Reference
SkQ1	HT1080 cells	40 nmol/L in culture medium	(1) Suppressed cell growth and prolonged cell mitosis (2) Induced distribution and activation of Aurora family kinases	[132]
	Tumor cells in culture or mouse models	40 nmol/L in culture medium; 250 nmol/kg∗bodyweight	(1) Decreased cell growth and the weight of subcutaneous tumors (2) Prolonged cell mitosis and apoptosis	[132]
	p53(-/-) mice	5 nmol/kg∗bodyweight per day	(1) Delayed appearance of tumors (2) Inhibited the growth of xenografts tumors and angiogenesis	[133]
	BALB/c mice in SPF environment	1 and 30 nmol/kg∗bodyweight per day	(1) Decreased the incidence of spontaneous cancers at the dosage of 30 nmol/kg∗bodyweight (2) Suppressed the cancer dissemination at 1 nmol/kg∗bodyweight dosage	[134]
	Benzopyrene-induced carcinogenesis in SHR mice	5 and 50 nmol/kg∗bodyweight per day	(1) Inhibited tumor growth (2) Dose-dependent effects were observed	[135]

KRSH	HeLa and MCF-7 cells	50 nmol/L in culture medium	(1) Inhibited greater tumor cell growth than the normal cells (2) Increased apoptosis of HeLa and MCF-7 cells, but not of MCF10A cells (3) Accumulated in mitochondria and increased mitochondrial depolarization	[136]

Mito-TEMPO	N-Nitrosodiethylamine-induced hepatocarcinogenesis in BALB/c mice	0.1 mg/kg∗bodyweight weekly	(1) Increased animal survival ratio and decreased tumor incidence and tumor multiplicity (2) Rescued the gap junctions and gap junctional intercellular communication of tumor cells	[137]

Notes: HeLa cells: cervical cancer cell line taken from Henrietta Lacks; HT1080: human sarcoma cell line; MCF-7: breast cancer cell line that consisted of the acronym of Michigan Cancer Foundation-7; p53: tumor protein p53; SPF; specific pathogen free.

## Data Availability

No data were used to support this review article.

## Additional Points

*Noteworthy Questions*. (1) What is the decisive mechanism in the development of mitochondria-targeted disease? (2) What is the most effective antioxidant for regulating the decisive mechanism (according to the experiment on separated mitochondria)? (3) Which delivery system will be the best choice for the mitochondria-targeted delivery of antioxidants? (4) How to optimize the effective MTA dosage in different administration approaches (e.g., oral, intravenous, or subcutaneous) for disease treatment? (5) How to standardize a clinical trial (e.g., how many patients to get involved, how long for patient tracking, and what parameters to assess side effects) for the evaluation of MTAs in disease treatment?
